# Near-field radiative heat transfer between topological insulators via surface plasmon polaritons

**DOI:** 10.1016/j.isci.2021.103408

**Published:** 2021-11-09

**Authors:** Ruiyi Liu, Lixin Ge, Biyuan Wu, Zheng Cui, Xiaohu Wu

**Affiliations:** 1 Institute of Advanced Technology, Shandong University, Jinan 250061, China; 2 Shandong Institute of Advanced Technology, Jinan 250100, China; 3 School of Physics and Electronic Engineering, Xinyang Normal University, Xinyang 464000, China; 4 School of Automation and Information Engineering, Xi'an University of Technology, Xi'an 710048, China

**Keywords:** Heat transfer, Applied sciences, Surface science

## Abstract

Recently, thanks to its excellent opto-electronic properties, two-dimension topological insulator not only has attracted broad interest in fields such as tunable detectors and nano-electronics but also shall yield more interesting prospect in thermal management, energy conversion, and so on. In this work, the excellent near-filed radiative heat transfer (NFRHT) resulting from monolayer topological insulator (Bi_2_Se_3_) is demonstrated. The NFRHT of this system is mainly dominated by the strong coupling effect of the surface plasmon polaritons (SPPs) between two Bi_2_Se_3_ sheets. Moreover, the non-monotonic dependence of the Fermi energy of Bi_2_Se_3_ on NFRHT is then discovered. It is indicated that the system can provide great thermal adjustability by controlling the Fermi energy, achieving a modulation factor of heat flux as high as 98.94%. Finally, the effect of substrate on the NFRHT is also explored. This work provides a promising pathway for the highly efficient thermal management.

## Highlights

• The excellent near-field radiative heat transfer of Bi_2_Se_3_ is demonstrated
• The influence of Fermi energy of Bi_2_Se_3_ on the heat flux is revealed
• The effect of substrate on the heat flux is explored

## Graphical abstract

**Figure:**
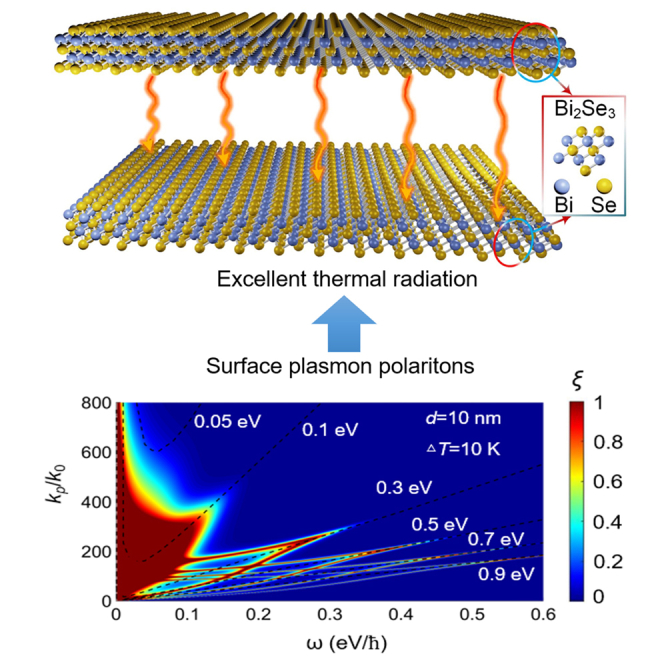

## Introduction

When two objects are brought to the micro/nano scale separations, the near-field radiative heat transfer (NFRHT) can exceed the Stefan-Boltzmann law of black-body radiation by several orders of magnitude (Polder and Van, 1971; Joulain et al., 2005; Kim et al., 2015; Biehs et al., 2010; Cuevas and Garcia-Vidal, 2018), due to the tunneling effect of evanescent modes. The huge energy flux of the NFRHT has received extensive interest and opens the door to next-generation energy control and conversion technologies (Zhang, 2020), including near-field thermophotovoltaics (Ilic et al., 2012a; Koyama et al., 2019; St-Gelais et al., 2017; Mittapally et al., 2021), photonic transformer (Zhao et al., 2021), noncontact thermal management (Papadakis et al., 2019; Ito et al., 2017; Otey et al., 2010; Ben-Abdallah and Biehs, 2014), and electroluminescent cooling (Zhu et al., 2019; Chen et al., 2015). Many researches have been devoted to explore new materials to obtain greater heat flux, thereby improving the performance of the above applications (Wu, 2021; Tang et al., 2020; Wu, Fu, and Zhang, 2018, 2020; Liu et al., 2017; Francoeur et al., 2011; Wu and Fu, 2021a, 2021b; Zhou et al., 2020; Shi et al., 2017; Li et al., 2021; Hu et al., 2021). For instance, the metamaterials, two-dimensional (2D) materials such as graphene (Ferrari et al., 2006), black phosphorus (Li et al., 2014), transition metal dichalcogenides, and hexagonal boron nitride (Laturia et al., 2018), have ignited a surge of researches, because of their outstanding optoelectronic properties, such as large spin-orbit coupling effect, ultrahigh charge carrier mobility, topological effect, and the complex interactions between light and matter. Compared with the conventional surface polaritons in polar dielectrics materials or metals, surface modes of 2D materials can exhibit extraordinary levels of light-matter interaction (Lai et al., 2021). Because of the exciting properties, the 2D materials are completely different from their parental materials, providing new pathway for nanoscale photonics (Hu et al., 2020). Recently, it was demonstrated that the surface plasmon polaritons (SPPs) or surface phonon polaritons, supported by 2D materials, can enhance observably the photon tunneling between two objects, thereby improving the performance of the NFRHT (Wu and Liu, 2020; Yan et al., 2013; Fang et al., 2014; Ilic et al., 2012b; Liu et al., 2021; Peng et al., 2015). Meanwhile, the analyzing of the hybrid effect between 2D materials and metamaterials (or dielectric substrates) on NFRHT has been greatly promoted (Lim et al., 2018). However, to the best of our knowledge, the study of NFRHT between 2D bismuth-based topological insulators (Bi_2_Se_3_) has not been conducted yet.

In recent years, bismuth selenide (Bi_2_Se_3_), a topological insulator, has received considerable interest, owing to its nontrivial surface states caused by the inverted band structure. Remarkably, the electrons occupying the surface states exhibit spin-momentum locking, which ensures the surface state has a higher fatigue resistance and a longer lifetime (Zhang et al., 2009). These nontrivial properties of the surface states hold a great promise in tunable THz detectors (Viti et al., 2016), emitters (Luo et al., 2013), lasers (Chen and Segev, 2021), and quantum computing (Paudel, and Leuenberger, 2013). As a novel Dirac plasmonic material, the Bi_2_Se_3_ can support a stronger SPP mode, which can be observed comparable with the noble metals (Politano et al., 2017). As a result, the Bi_2_Se_3_ could be an excellent candidate for noncontact thermal management. Therefore, it is imperative to understand how the surface plasmon polaritons arouses noncontact heat exchange in topological insulator at the near-field.

In this work, the NFRHT for Bi_2_Se_3_ sheets is investigated theoretically. Owing to the excellent surface states of Bi_2_Se_3_, the NFRHT performance of Bi_2_Se_3_ can greatly exceed that from other traditional plasmon materials. In addition, we study the variation of NFRHT with the vacuum gap and the Fermi energy of Bi_2_Se_3_. By tuning the Fermi energy of Bi_2_Se_3_, a modulation factor of heat flux as high as 98.92% is revealed. Moreover, we examine the interference effect of substrate. The spectral heat flux, photonic transmission coefficient, as well as the plasmon dispersion relations are analyzed to understand the physical mechanism of the excellent thermal performance of Bi_2_Se_3_. Our work not only reveals the unique advantages of Bi_2_Se_3_ in radiation heat transfer but also paves a new way to its application in nano-scale thermal modulation.

## Results

**Figure 1:**
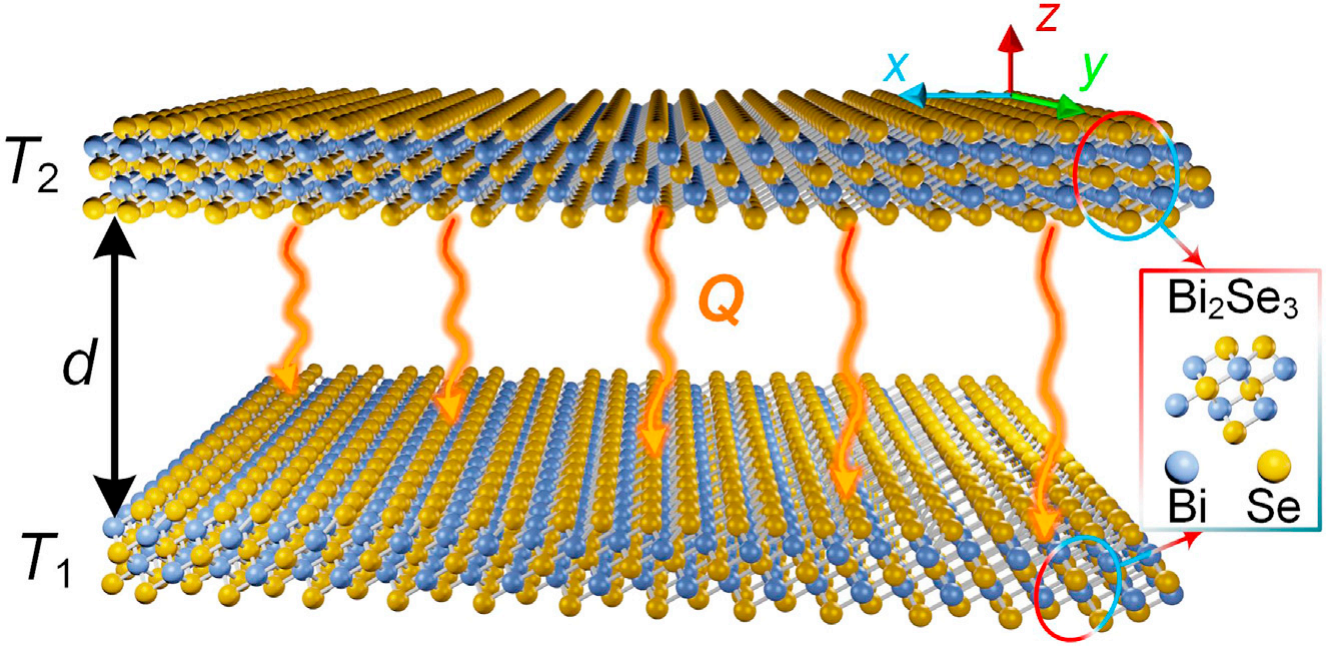
Schematic of near-field radiative heat transfer between two sheets of Bi_2_Se_3_ The temperature of the top sheet and the bottom one is *T*_1_ (300 K) and *T*_2_ (310 K), respectively.

Let us consider a system composed of two monolayer Bi_2_Se_3_ sheets as sketched in Figure 1, setting the vacuum gap as *d*. The monolayer Bi_2_Se_3_ sheet possesses a P-3m1 space group (No. 164). For Bi atoms, each Bi atom connects with six adjacent Se atoms and forms Bi-Se bonds. Similarly, six Se-Bi bonds are formed around the Se atom (Zhang et al., 2020). The monolayer Bi_2_Se_3_ sheet is modeled with a sheet conductivity, *σ*, and the variations of *σ* with the Fermi energy *E*_*f*_ can be seen in Figure S1. The temperature of the top sheet and the bottom one is set to *T*_1_ = 300 K and *T*_2_ = 310 K, respectively. According to the set parameters, we can calculate the NFRHT between two parallel topological insulator sheets.

### Enhancement effect of NFRHT

**Figure 2:**
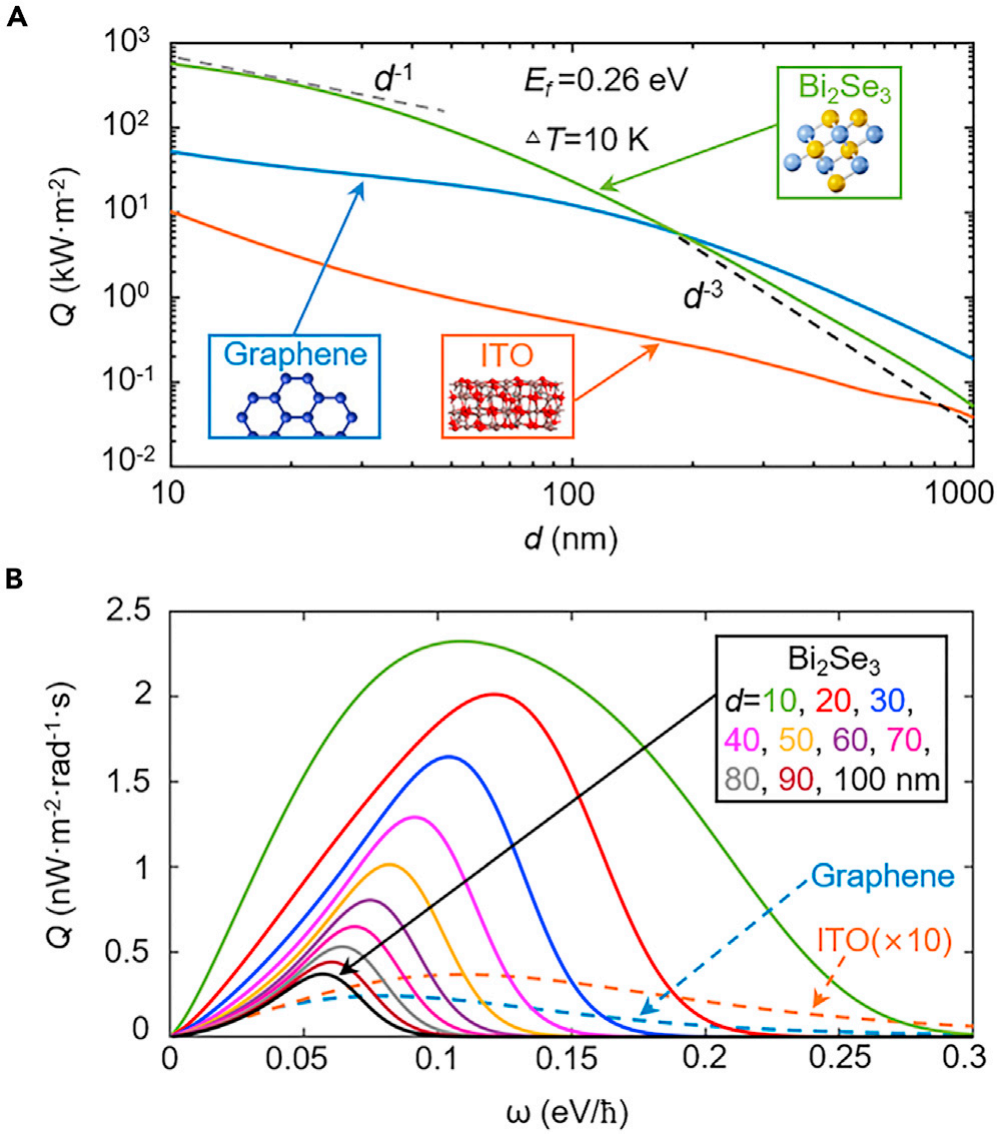
Heat flux varing with vacuum gaps (A–B) (A) Heat flux *Q* of the monolayer Bi_2_Se_3_ sheet, monolayer graphene sheet, and bulk ITO, with different vacuum gaps. (B) Spectral heat flux *Q* (*ω*) between two sheets of Bi_2_Se_3_ under different vacuum gaps. For comparison, the spectral heat flux *Q* (*ω*) between two graphene sheets and two bulk ITO with *d* = 10 nm is given in the dashed lines. The Fermi energies of Bi_2_Se_3_ and graphene are both 0.26 eV.

First, we assume the two topological insulator sheets (thickness at atomic scale) are both in suspended state, i.e., the dielectric substrate is assumed to be vacuum, and the permittivity of the dielectric substrate is *ε*_*s*_ = 1. The range of vacuum gap *d* is set between 10 and 1,000 nm. The Fermi energy is fixed as *E*_*f*_ = 0.26 eV. Figure 2A shows the huge heat flux between two Bi_2_Se_3_ sheets as a function of separation distance. For comparison, the radiative heat flux of two representative plasmon materials, graphene sheets and indium tin oxide (ITO), are also plotted. The optical parameters of ITO and graphene can be modeled in Zhao et al. (2017) and Song and Cheng (2016). In this work, the Fermi level of graphene is also set as 0.26 eV, whereas the plasma frequency of ITO is fixed at 0.5 eV/*ћ*.

It can be seen clearly that the NFRHT between two Bi_2_Se_3_ sheets has been enhanced greatly for nanoscale separation and its magnitude can be much higher than the black-body limit. Note that the radiative heat flux of the black body is given by $Q_{bb}\left(T_{1},T_{2}\right)=\sigma_{\mathrm{SB}}\left(T_{2}^{4}-T_{1}^{4}\right)\approx 64.37\text{W}\cdot {\text{m}}^{-2}$, where $\sigma_{\mathrm{SB}}$ is the Stefan-Boltzmann constant. Moreover, the heat flux between Bi_2_Se_3_ sheets can outperform those of the monolayer graphene and bulk ITO, especially at small gap distances. At the vacuum gap *d* of 10 nm, the radiative heat flux between Bi_2_Se_3_ sheets can yield 573.04 kW·m^−2^, which is more than ten times that of monolayer graphene (52.64 kW·m^−2^) and fifty times that of bulk ITO (10.32 kW·m^−2^). However, as the gap size increases, the radiative heat flux of the monolayer Bi_2_Se_3_ sheet decreases monotonically due to a rapid decay of the local density of states. When the vacuum gap is gradually pushed to a larger scale, i.e., above 10 nm, the NFRHT decreases quickly and exhibits the well-known law of *d*^−1^ (represented by the gray dashed line), as observed by Pablo et al. for 2D material (Pablo et al., 2015). It is worth noting that the tendency of NFRHT for the monolayer Bi_2_Se_3_ gradually varies to *d*^−3^ (denoted by the black dashed line) at vacuum gap sizes around 10^2^–10^3^ nm. As can be seen, when the vacuum gap approaches 1,000 nm, the radiative heat flux between Bi_2_Se_3_ sheets exhibits a lower value of 51.22 W·m^−2^, which is 0.28 times that between graphene monolayers (186.25 W·m^−2^). For more analysis on the variation trend of *Q* with *d*, see Figure S2.

Figure 2B shows the spectral heat flux between two sheets of Bi_2_Se_3_ with various vacuum gaps; for comparison, the results of two graphene sheets and two bulk ITO with *d* = 10 nm are also given. Unsurprisingly, with the increase of vacuum gap, the radiative heat flux reduces. In addition, the peak of the spectral radiative heat flux is redshifted from 0.12 eV/*ћ* for *d* = 20 nm to around 0.06 eV/*ћ* for *d* = 100 nm. This redshift of the spectral radiative heat flux can be explained by the attenuation length (*δ* = 1/Im(*k*_z_)), that is, the surface polaritons located at high frequencies are more easily filtered by lager vacuum gaps (Liu and Zhang, 2015). In addition, when the vacuum gap is 10 nm, the spectral heat flux between Bi_2_Se_3_ sheets is higher than that of graphene and ITO, resulting in a more significant heat flux shown in Figure 2A.

**Figure 3:**
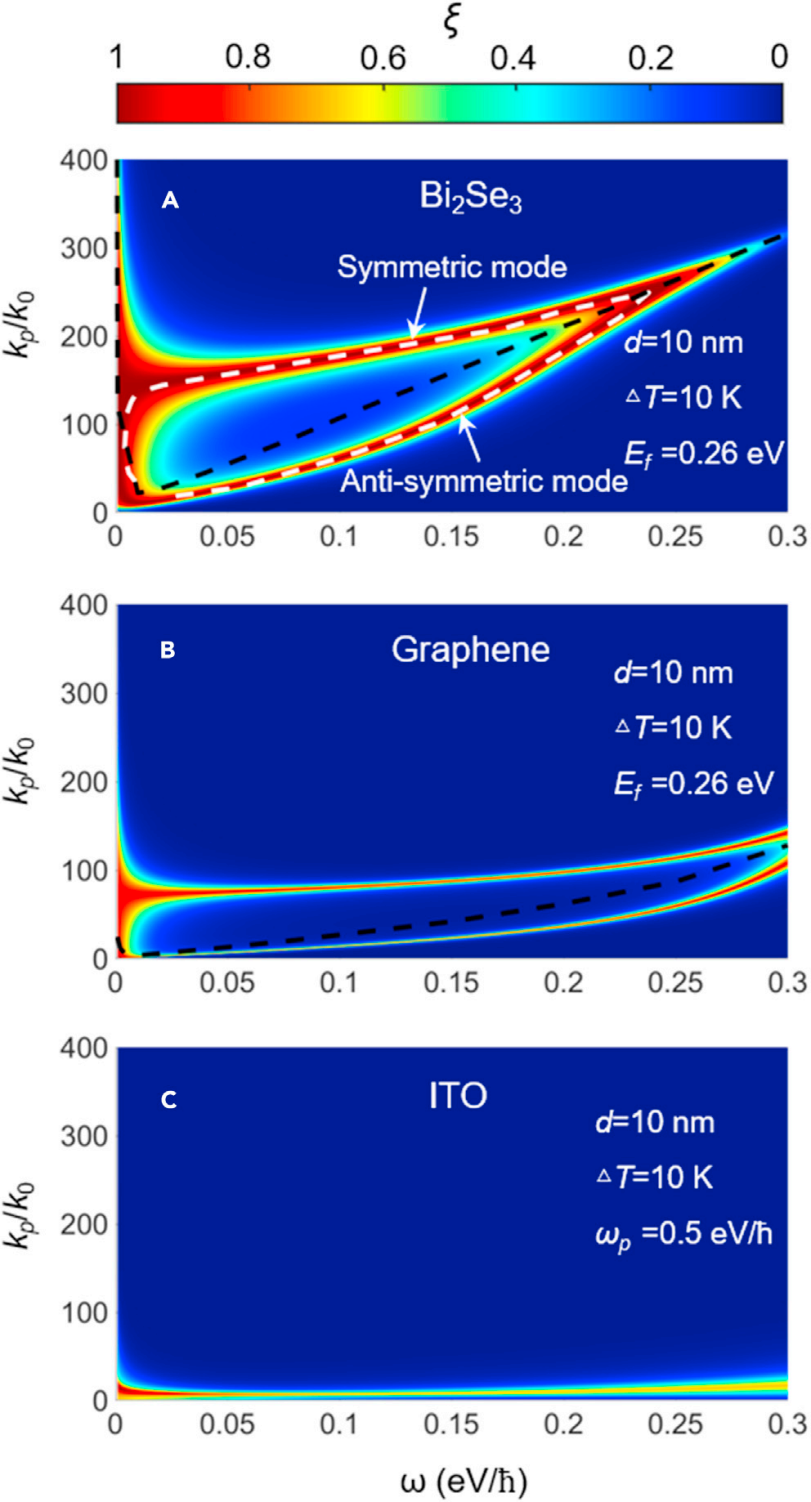
The photonic transmission coefficient (PTC) for different structures (A–C) (A) Two Bi_2_Se_3_ sheets, (B) two graphene sheets, and (C) two semi-infinite slabs of ITO. The black dashed lines represent the dispersion relations, and the white dashed lines represent the anti-symmetric and symmetric modes of SPPs. The vacuum gap is *d* = 10 nm.

The physical mechanism of the great enhancement of radiative heat flux can be understood by analyzing the photonic transmission coefficient (PTC), as shown in Figure 3. The bright bands in the figure indicate the tunneling probability of thermally excited photons excited by different polaritons, which is the major contributor to the high near-field heat flux. For the Bi_2_Se_3_, since it can support polariton modes of high density, there is a bright and strong branch of PTC. In addition, since both the lower and upper vacuum-Bi_2_Se_3_ interfaces support evanescent waves that decay exponentially along the direction perpendicular to the interface, the evanescent field of SPPs associated with each interface can interact with each other, leading to a splitting of the single resonance mode into antisymmetric (at small wavevector region) and symmetric (at high wavevector region) modes in Figure 3A. The white lines represent the dispersion relation of antisymmetric and symmetric SPPs, which can nicely predict the maximum of the PTC. To further confirm the role of SPPs of Bi_2_Se_3_ in the NFRHT, the plasmon dispersion relations (black dashed line) of the Bi_2_Se_3_ sheet are plotted in Figure 3A. The black curve nicely locates at the middle of the bright branch, which explicitly verifies the dominance of SPPs in NFRHT. For comparison, the PTC between two graphene sheets (with a similar Fermi energy) is shown in Figure 3B, where its bright band is narrower. In addition, it can be seen that the SPPs of Bi_2_Se_3_ can hold a higher wavevector range compared with graphene. This is also the main reason why the NFRHT of graphene is weaker than that of Bi_2_Se_3_. When the configuration is replaced by ITO, it can be seen that the wavevector of the PTC is greatly suppressed in Figure 3C.

## Discussion

### The role of Fermi energy in NFRHT

Note that one important aspect of the optical behavior for Bi_2_Se_3_ is the dependence of the conductivity on the value of Fermi energy. The value of Fermi energy of Bi_2_Se_3_ can be modulated by the doping carrier concentration, which can be controlled either chemically or electrically by introducing anion during fabrication. Among them, the relationship between Fermi energy and electron doping can be expressed as$E_{f}=\hbar v_{f}\sqrt{2\pi n_{i}}$ (Wang et al., 2020). In addition, from a practical viewpoint, since the dynamic modulation of thermal radiation always is a challenging and considerable topic in thermal engineering, it is indispensable to consider the active modulation of the Fermi energy on the NFRHT.

**Figure 4:**
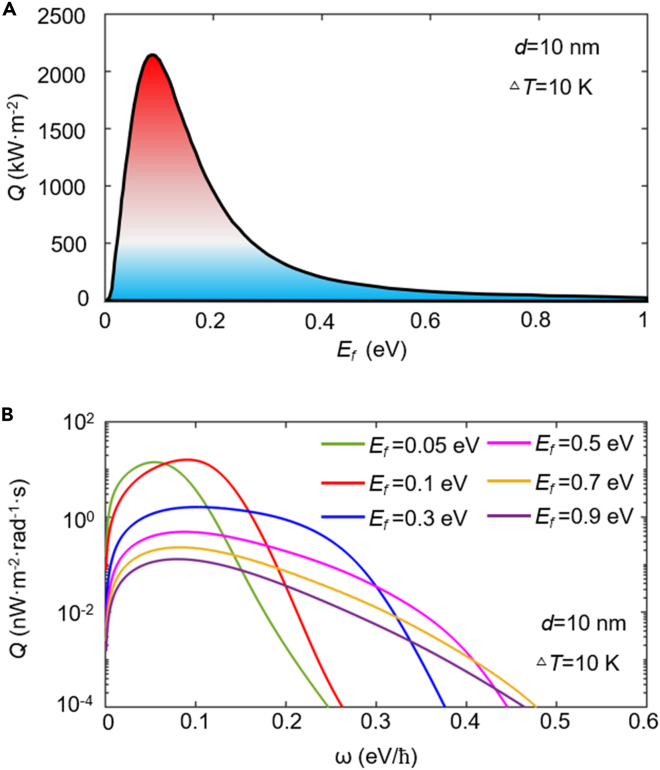
Heat flux varing with Fermi energy (A–B) (A) The total heat flux between two Bi_2_Se_3_ sheets varies with the Fermi energy. (B) Spectral heat flux *Q* (*ω*) under different Fermi energies.

The heat fluxes for the topological insulator system with different Fermi energies at *d* = 10 nm are shown in Figure 4A (the calculation results under other different vacuum gaps can be seen in Figure S3). We first observe that the heat flux reveals a non-monotonic dependency versus the Fermi energy. One can further see that, as the Fermi energy increases, the heat flux first reaches to a higher value. The optimal thermal effect of the system is observed at *E*_*f*_ = 0.09 eV, which implies that the optical properties of Bi_2_Se_3_ with this electronic structure are more suitable for NFRHT. The maximum heat flux can reach 2,155.24 kW·m^−2^. With the further increase of Fermi energy, the heat flux of the system drops drastically in Figure 4A. We can see the heat flux quickly attenuate to 22.88 kW·m^−2^ at 1 eV, and the modulated factor (1-*Q*(*E*_*f*_)/Q_max_) is as high as 98.94%. To further explain the role of Fermi energy, in Figure 4B, we plot the spectral heat flux with *E*_*f*_ = 0.05, 0.1, 0.3, 0.5, 0.7, and 0.9 eV, respectively. For *E*_*f*_ = 0.05 eV, the value of peak is 14.29 nW·m^−2^·rad^−1^·s at 0.055 eV/*ℏ*. For a higher Fermi energy of *E*_*f*_ = 0.1 eV, the spectral heat flux gets blue-shift and the maximum increases to 16.08 nW·m^−2^·rad^−1^·s. While the Fermi energy further increases, the spectral bandwidth of the spectral heat flux gets broader, but the value of peak reduces drastically. For *E*_*f*_ = 0.9 eV, the value of peak is only 0.13 nW·m^−2^·rad^−1^·s at 0.08 eV/*ℏ*.

**Figure 5:**
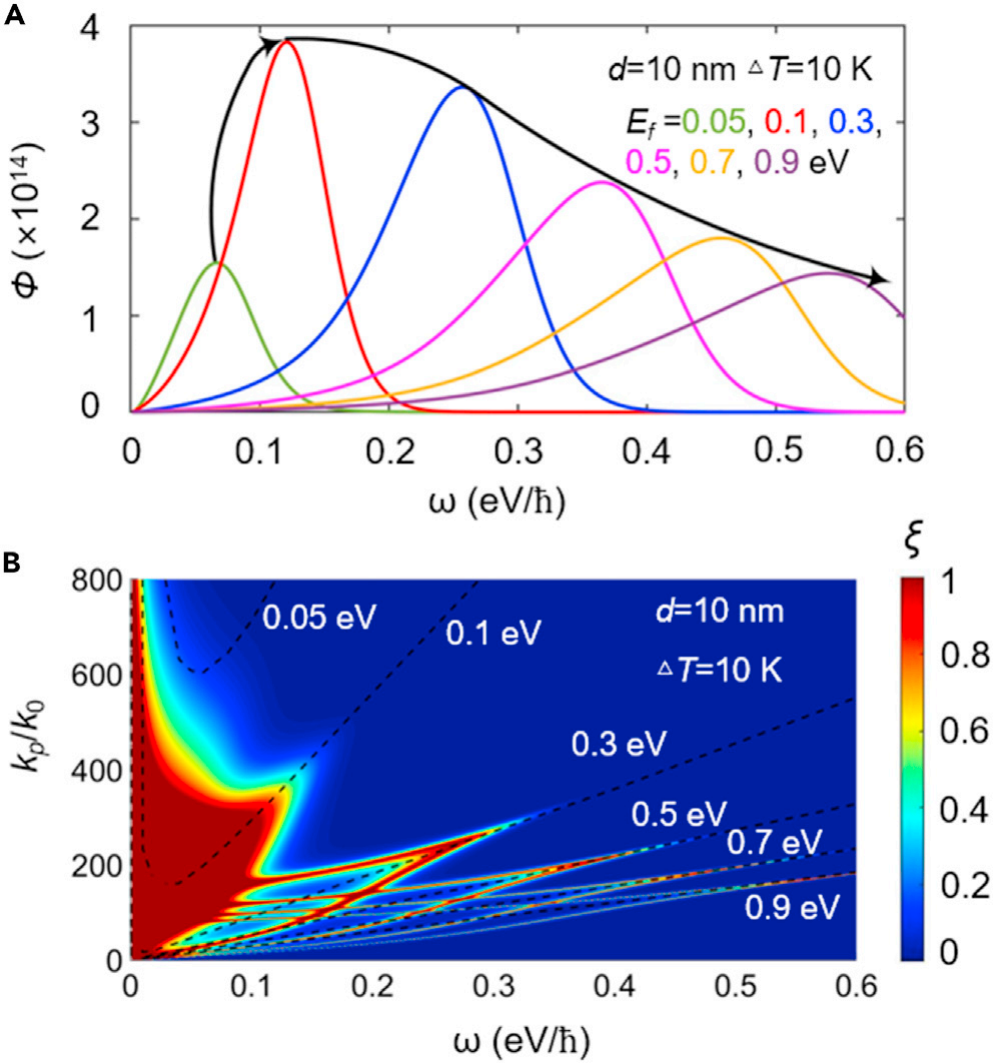
Analysis of energy transfer characteristics at different Fermi energies (A–B) (A) Contour plot of energy transfer function *Φ(ω)*, as a function of frequency with different Fermi energies. (B) The photonic transmission coefficient for different Fermi energies. The black dashed line in (B) represents the dispersion relations of the monolayer Bi_2_Se_3_ with different Fermi energies.

To visualize the radiative heat flux varying with the Fermi energy, in Figure 5A, we calculate the energy transfer function$\Phi =Q\left(\omega \right)/\left(\Theta \left(\omega ,T_{2}\right)-\Theta \left(\omega ,T_{1}\right)\right)$. While the Fermi energy increases from 0.05 to 0.1 eV, the maximum of energy transfer function generated by surface polaritons increases drastically from 1.54×10^14^ at 0.067 eV/*ℏ* to 3.83×10^14^ at 0.12 eV/*ℏ*, which corresponds to the increasing spectral heat flux in Figure 4B at the lower Fermi energy range. As the Fermi energy further increases, the SPPs are excited strongly only by high photonic energy; this also explains the blue-shifted peak of energy transfer function shown in Figure 5A. However, the contributions of SPPs to NFRHT at high frequencies are negligible, owing to the mean energy of harmonic oscillator of Plank decay exponentially with frequency at this temperature. Therefore, in Figure 4B, when the Fermi energy is above 0.5 eV, it is difficult to observe the peak of the spectral heat flux caused by SPPs.

In Figure 5B, we show the PTCs of Bi_2_Se_3_ with different Fermi energies. We can observe that two branches of SPPs gradually move to higher wavevector and lower frequency with the decreasing Fermi energy. Meanwhile, with the decrease of Fermi energy, the symmetrical and anti-symmetric branches gradually become stronger and brighter. Eventually, when the Fermi energy decreases to 0.1 eV, the PTC forms a continuous region with a near-unity value in the *ω-k*_*ρ*_ phase space, hence resulting in a large increase in the heat flux. However, as the Fermi energy further decreases to 0.05 eV, it can be seen that the coupling effect between Bi_2_Se_3_ sheets becomes so weak that it is difficult to observe, hence resulting in a great recession of the heat flux in Figure 4A. To get insight into the mechanism of the above results, in Figure 5B, we exhibit the plasmon dispersion curves for different Fermi energies. Notice that, when the Bi_2_Se_3_ sheet holds a Fermi energy of 0.05 eV, its plasmon dispersion is always in the higher wavevector range (>600*k*_0_), which greatly weakens the attenuation length of surface waves. Thereby, compared with other Fermi energies, the SPPs for *E*_*f*_ = 0.05 eV is easily filtered by the vacuum. For larger Fermi energies, since the behavior of the Bi_2_Se_3_ sheet with higher doping concentration is closer to that of metal, the Bi_2_Se_3_ with a higher doping concentration has a higher resonant frequency of SPPs. Therefore, with the increase of *E*_*f*_, we find that the plasmon dispersions dramatically transfer to a lower wavevector, hence decreasing the NFRHT. It can be seen that the branches of PTC in Figure 5B is consistent with our observation about the plasmon dispersion in the above analysis.

### The interference effect of substrate on NFRHT

**Figure 6:**
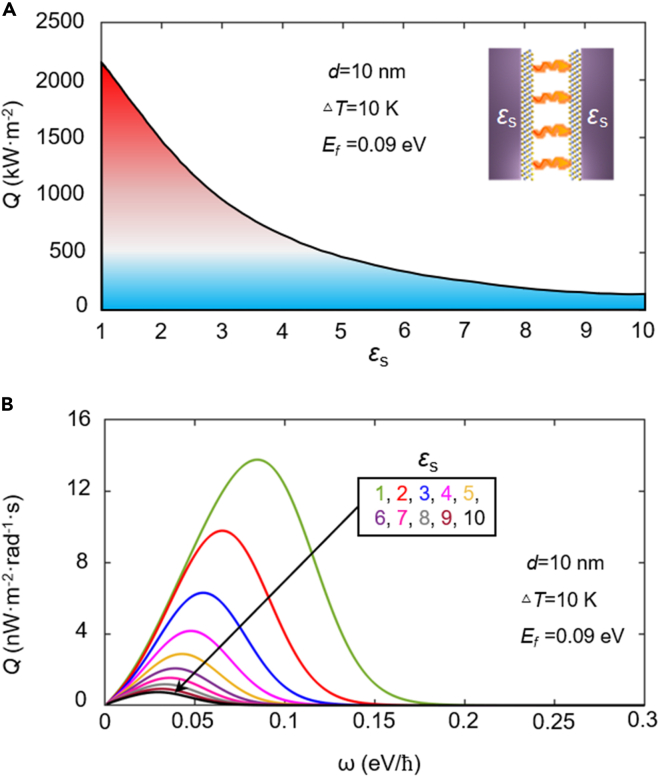
Heat flux varing with the permittivity of substrate (A–B) (A) Radiative heat flux between two sheets of Bi_2_Se_3_ deposited on substrates with different permittivity. (B) Spectral heat flux under different dielectric substrates. The vacuum gap is *d* = 10 nm. The Fermi energy is 0.09 eV.

Technically, the suspended 2D-material sheets can be realized in the experiments. However, it is difficult to use this suspension technology in application for thermal controls. In reality, 2D-material sheets are generally deposited on a variety of substrates (Yang et al., 2018). Therefore, the interference effect of substrate on the NFRHT of topological insulator is important for the experiments and various application development. The NFRHT of Bi_2_Se_3_ deposited on different substrates is demonstrated in Figure 6; the Fermi energy of Bi_2_Se_3_ is set to 0.09 eV (the calculation results at other different Fermi energies can be seen in Figures S4 and S5). For simplicity, the dielectric substrate is chosen as nonpolar and nondispersive materials. It is found that the heat flux of the system drops drastically as the permittivity of the dielectric substrate *ε*_*s*_ increases, e.g., from 2,155 kW·m^−2^ for *ε*_*s*_ = 1 to 133 kW·m^−2^ for *ε*_*s*_ = 10. To further explain the substrate effect, the spectral heat flux with different *ε*_*s*_ is shown in Figure 6B. It should be noticed that, with the increasing permittivity of the dielectric substrate, the peak of spectral heat flux undergoes an obvious redshift, for instance, the frequency of spectral peak gradually moves from 0.085 eV/*ℏ* for *ε*_*s*_ = 1 to 0.03 eV/*ℏ* for *ε*_*s*_ = 10. In addition, the amplitudes and bandwidth of spectral heat flux decrease accordingly, resulting in a decrease of total heat flux. In other words, in actual thermal management, to ensure the sufficient radiation heat transfer between two sheets of Bi_2_Se_3_, the permittivity of the dielectric substrate should be as small as possible.

**Figure 7:**
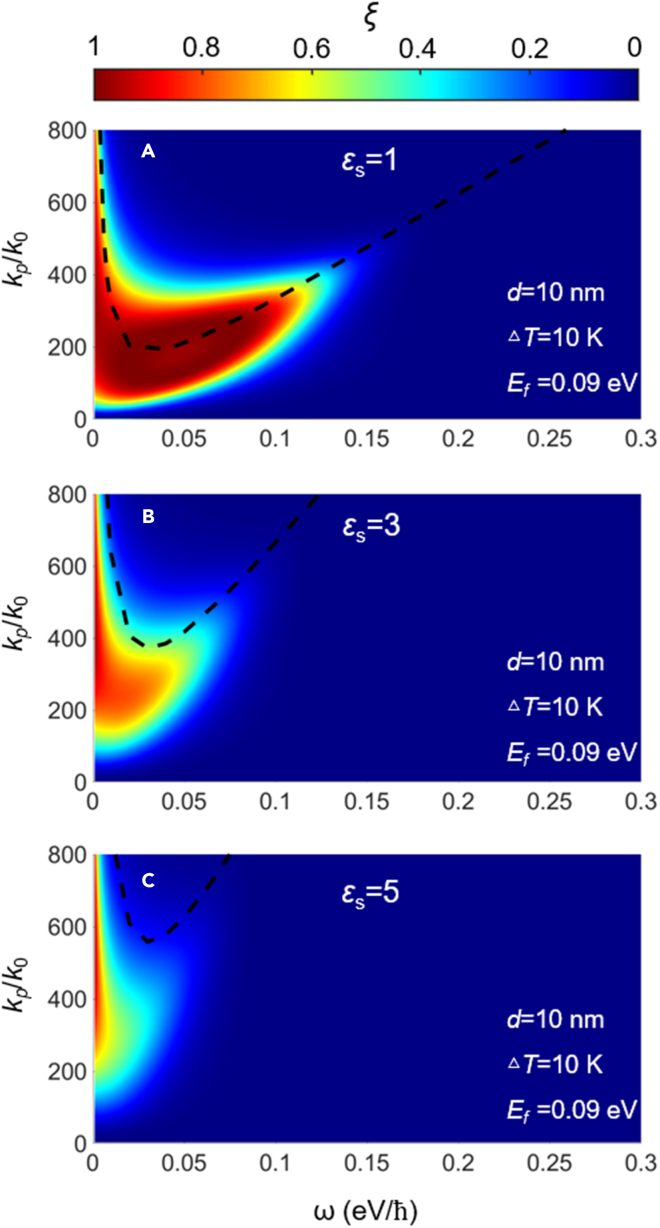
The photonic transmission coefficient for different dielectric substrates (A–C) (A) *ε*_*s*_ = 1, (B) *ε*_*s*_ = 3, and (C) *ε*_*s*_ = 5. The black dashed lines correspond to the dispersion relations of the coupled system between different dielectric substrates and Bi_2_Se_3_. The vacuum gap is *d* = 10 nm. The Fermi energy is 0.09 eV.

The origin of the dependency of the NFRHT on the interference effect of substrate can be explored with a detailed exhibition of the PTC and dispersion relations of the coupled system. In particular, we show PTC with dielectric substrates of *ε*_*s*_ = 1, *ε*_*s*_ = 3, and *ε*_*s*_ = 5, respectively, in Figures 7A–7C. First, we discuss the case of *ε*_*s*_ = 1, i.e., the Bi_2_Se_3_ sheets are in a suspended state. Compared with the case of *E*_*f*_ = 0.26 eV in Figure 3A, as *E*_*f*_ decreases to 0.09 eV, the symmetric and anti-symmetric branches of SPPs in Figure 7A hold a stronger state, so the division of branches disappears, and a continuous region with a near-unity value in the *ω-k*_*ρ*_ phase space is formed. In addition, as seen in Figure 7A, the SPPs hold a larger wavevector range than that in Figure 3A, thus the total heat flux increases, as shown in Figure 4A. It can be seen that the dispersion relation line of the coupled system composed with Bi_2_Se_3_ and dielectric substrates is located exactly at the middle of the bright branch.

However, the intense coupling effect of evanescent waves between two Bi_2_Se_3_ sheets would not be maintained when the substrates are considered, as shown in Figures 7B and 7C. The maximal value of PTC gradually decreases from 1 for *ε*_*s*_ = 1 to 0.7 for *ε*_*s*_ = 5. Among them, at the high frequency (about 0.09 eV/*ℏ*), as the permittivity of the dielectric substrate increases, it is difficult to observe the bright branch excited by SPPs in Figure 7C. This is also the main reason for the sharp decline in NFRHT with the increasing permittivity of the dielectric substrate in Figure 6A. With the increasing permittivity of the dielectric substrate, the bright band of PTC gradually shrinks toward the lower frequency range, thereby producing a decreasing bandwidth of spectral heat flux in Figure 6B. Moreover, owing to the interference effect of substrate, the dispersion relation line of the coupled system gradually transits to a higher wavevector range with the increasing permittivity of the dielectric substrate, as shown in Figure 7. Meanwhile, the mismatch between the dispersion relation and bright branch gradually appears, and the bright bands are all located below the dispersion curve. This is because, as the wavevector of the dispersion curve increases with permittivity, the attenuation length of the symmetric mode of SPPs above the dispersion curve decreases sharply. At the higher wavevector region, the evanescent wave dominated by symmetric mode is easily filtered by the middle gap and no longer stimulates obvious SPPs coupling effect between two Bi_2_Se_3_ sheets.

### Conclusions

In summary, the excellent NFRHT of the monolayer bismuth-based topological insulator (Bi_2_Se_3_) is investigated. The high performance of Bi_2_Se_3_ on the enhancement of NFRHT has been demonstrated in comparison with other traditional plasmon materials. For a nanoscale separation of 10 nm, the heat flux of Bi_2_Se_3_ sheets can be more than ten times that of monolayer graphene and fifty times for bulk ITO. The great enhancement of NFRHT is induced by the SPPs in Bi_2_Se_3_. Moreover, a non-monotonic dependency between the NFRHT and the Fermi energies has been clearly demonstrated. The underlying mechanism is mainly ascribed to the transition of SPP dispersion toward higher frequencies and lower wave vectors with increasing Fermi energy. It is also shown that Bi_2_Se_3_ can provide a modulation factor greater than 98.94% by controlling Fermi energy. Finally, the substrate effect on the NFRHT of Bi_2_Se_3_ is analyzed. With the increase of the permittivity of dielectric substrate, the amplitude and bandwidth of spectral heat flux will decrease, resulting in the decrease of heat flux; this phenomenon is explained by analyzing the photonic transmission coefficient and the dispersion relations. This work not only reveals the NFRHT of bismuth-based topological insulator but also paves a way to nanoscale thermal modulation based on this kind of new material.

### Limitations of the study

In this work, we numerically simulate the excellent NFRHT of Bi_2_Se_3_. However, the effect of surface states of Bi_2_Se_3_ on radiative heat transfer still needs further experimental observation. In the study of substrate effect, we chose nonpolar and nondispersive material as the dielectric substrate. When choosing polar materials as substrates, the polariton hybrid effect induced by the substrate needs to be further explored. Moreover, in this work we only discuss the NFRHT of Bi_2_Se_3_ sheets; the other structures, such as Bi_2_Se_3_ grating structures, may produce new phenomena in radiation heat transfer and play a role in future applications.

## STAR★Methods

### Key resources table

**Table:** 

REAGENT or RESOURCE	SOURCE	IDENTIFIER
**Software and algorithms**		

MATLAB R2019b	This paper	N/A

### Resource availability

#### Lead contact

Further information and requests for resources and reagents should be directed to and will be fulfilled by the lead contact, Dr. Xiaohu Wu (xiaohu.wu@iat.cn).

#### Materials availability

This study did not generate new unique materials.

### Method details

The computational analysis presented in this study was performed in the software MATLAB R2019b. The monolayer Bi_2_Se_3_ sheet is modeled with a sheet conductivity, *σ*, that includes the contributions from both the interband and intraband transitions. Due to the electronic properties of Bi and Se atoms, monolayer Bi_2_Se_3_ shows different carrier density and mobility from graphene and ITO, resulting in different conductivity. The conductivity of Bi_2_Se_3_ can be written as (Wang et al., 2020):

(Equation 1) $$\sigma =\frac{e^{2}}{\hbar^{2}}\frac{E_{f}}{4\pi }\frac{i}{\omega +i\tau^{-1}}$$

(Equation 2) $$\tau =\frac{\mu_{m}E_{f}}{ev_{F}^{2}}$$

where *τ* is the relaxation time, *E*_*f*_ is the Fermi energy of Bi_2_Se_3_, depending on the carrier concentration, the Fermi energy of Bi_2_Se_3_ could be close to 0 eV, and the minimum value we consider in this paper is 0.05 eV. The sheet of Bi_2_Se_3_ can grow on a (Bi_0.5_In_0.5_)_2_Se_3_ buffer layer on *c*-plane sapphire. Correspondingly, the mobility is given as *μ*_*m*_ ≈ 600 cm^2^ V^−1^ s^−1^, and the Fermi velocity is *v*_*F*_ ≈ 5×10^5^ m/s. Figure S1 shows the real and imaginary parts of *σ* with different Fermi energies, the values are normalized by $\sigma_{0}=e^{2}/4\hbar$. The curves in Figure S1 are identical with the prediction from Equation (1). As can be seen from Figure S1B, a small imaginary part means a small dissipation of surface states, so the heat flux of Bi_2_Se_3_ is significantly enhanced compared with graphene and ITO.

According to the fluctuation electrodynamics and dyadic Green's function, the near-field radiative heat transfer (NFRHT) between two sheets of Bi_2_Se_3_ is given by (Chen et al., 2020):

(Equation 3) $$Q=\int_{0}^{\infty }Q\left(\omega \right)\cdot d\omega =\frac{1}{4\pi^{2}}\int_{0}^{\infty }d\omega \cdot \left[\Theta \left(\omega ,T_{2}\right)-\Theta \left(\omega ,T_{1}\right)\right]\cdot \int_{0}^{\infty }\xi \left(\omega ,k_{\rho }\right)\cdot k_{\rho }dk_{\rho }$$

where Θ and *ћ* are the mean energy of a Planck oscillator and the reduced Planck constant, respectively. The photonic transmission coefficient *ξ* represents the tunneling probability of thermal photons, which can be written as:

(Equation 4) ξ(ω,kρ)={(1−|rp,s|2−|tp,s|2)(1−|rp,s|2−|tp,s|2)|1−rp,s⋅rp,s⋅e2i⋅kz0⋅d|2,kρ≤ω/c4(Im(rp,s))(Im(rp,s))e−2Im(kz0)⋅d|1−rp,s⋅rp,s⋅e2i⋅kz0⋅d|2,kρ>ω/c

where *k*_*ρ*_, *k*_0_ = *ω/c*, and $k_{z0}=\sqrt{k_{0}^{2}-k_{\rho }^{2}}$ are the surface parallel wavevector, the wavevector in vacuum and the tangential wavevector perpendicular to the *x-y* plane in vacuum, respectively. When the surface parallel wavevector greater than the wavevector in vacuum, the electromagnetic wave excited by thermal energy is evanescent waves. *r* ^*p*,*s*^ is the reflection coefficient for *p* and *s* polarizations. In this study, atomic-scale topological insulators can be modeled as a conduction monolayer covering on a dielectric substrate, the reflection coefficients in Equation (4) can be commonly expressed as (Zhang et al., 2019):

(Equation 5) $$r^{p}=\frac{k_{z0}\cdot \varepsilon_{s}-k_{z}+\frac{\sigma k_{z0}k_{z}}{\omega \varepsilon_{0}}}{k_{z0}\cdot \varepsilon_{s}+k_{z}+\frac{\sigma k_{z0}k_{z}}{\omega \varepsilon_{0}}}$$

(Equation 6) $$r^{s}=\frac{k_{z0}-k_{z}-\sigma \mu_{0}\omega }{k_{z0}+k_{z}+\sigma \mu_{0}\omega }$$

in which *ε*_*s*_ is the permittivity of the dielectric substrate, *μ*_0_ is the permeability of vacuum, and $k_{z}=\sqrt{\varepsilon_{s}{\left(\omega /c\right)}^{2}-{k_{\rho }}^{2}}$ is the *z* component of wavevector in the substrate.

According to the plasmon dispersion relations, we plotted the dispersion curves in the photonic transmission coefficient diagrams for further analysis. The symmetric and anti-symmetric SPPs dispersion relations can be obtained by the following (Meyer et al., 2007):

(Equation 7) $$1-r^{p}\cdot r^{p}\cdot e^{2i\cdot k_{z0}\cdot d}=0$$

and the plasmon dispersion relations can be obtained by the following:

(Equation 8) $$\left(2\frac{k_{z0}}{k_{0}}+\sqrt{\frac{\mu_{0}}{\varepsilon_{0}}}\sigma \right)\left(2\frac{k_{0}}{k_{z0}}+\sqrt{\frac{\mu_{0}}{\varepsilon_{0}}}\sigma \right)=0$$

Here, it should be pointed out that in order to obtain the dispersion relationship of the coupled system with dielectric substrates, we need to modify Equation (8) as:

(Equation 9) $$\left(\frac{k_{z0}}{k_{0}}+\frac{k_{z}}{k_{0}}+\sqrt{\frac{\mu_{0}}{\varepsilon_{0}}}\sigma \right)\left(\frac{k_{0}}{k_{z0}}+\frac{\varepsilon_{s}k_{0}}{k_{z}}+\sqrt{\frac{\mu_{0}}{\varepsilon_{0}}}\sigma \right)=0$$

The Matlab code for calculating near-field radiative heat transfer can be found in Data S1.

## Data Availability

• The code used in this study can be obtained in the supplemental information • All data reported in this article will be shared by the lead contact upon request • Any additional information required to analyze the data reported in this study is available from the lead contact upon request.
