# Supplementary material for: Near-field radiative heat transfer between topological insulators via surface plasmon polaritons
Source: iScience. 2021 Nov 9;24(12):103408. doi: 10.1016/j.isci.2021.103408 (PMC8609043; doi:10.1016/j.isci.2021.103408)
Supplement: Document S1. Figures S1–S5 and Data S1 [file mmc1.pdf]

iScience, Volume *24*

Supplemental information

Near-field radiative heat transfer between topological insulators via surface plasmon polaritons

Ruiyi Liu, Lixin Ge, Biyuan Wu, Zheng Cui, and Xiaohu Wu

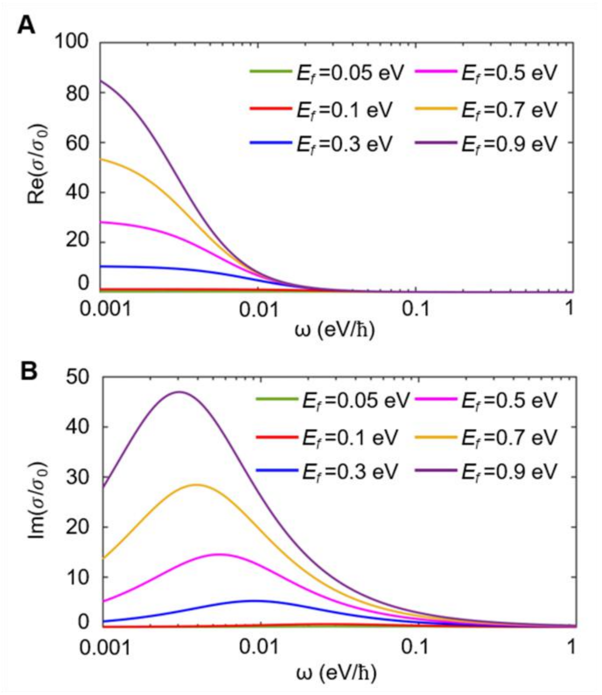

Figure S1. Sheet conductivity of Bi_2Se_3 with different Fermi energies: (A) real part and (B) imaginary part. The values are normalized by $\sigma_0 = e^2/4\hbar$. Related to STAR Methods.

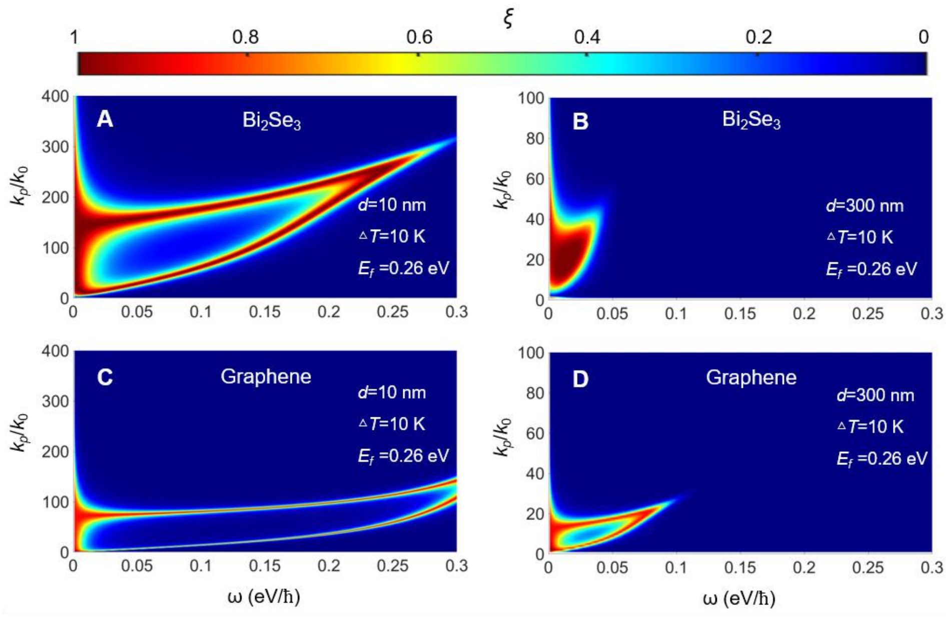

Figure S2. The PTC for two Bi_2Se_3 sheets when (A) d = 10 nm and (B) d = 300 nm, and for two graphene sheets when (C) d = 10 nm and (D) d = 300 nm. Related to Figure 2.

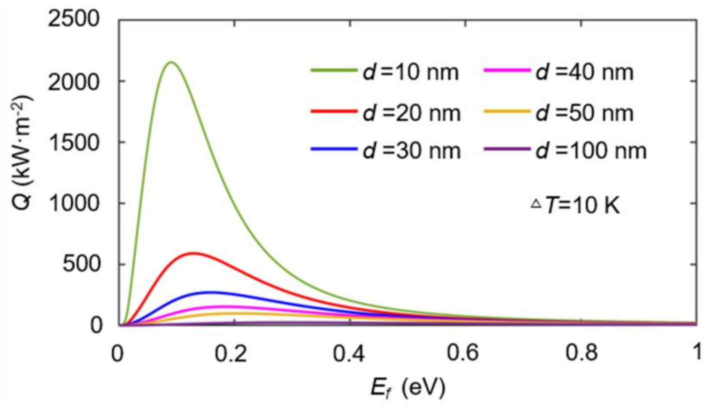

Figure S3. The total heat flux between two Bi_2Se_3 sheets at different vacuum spacing varies with the Fermi energy. Related to Figure 4.

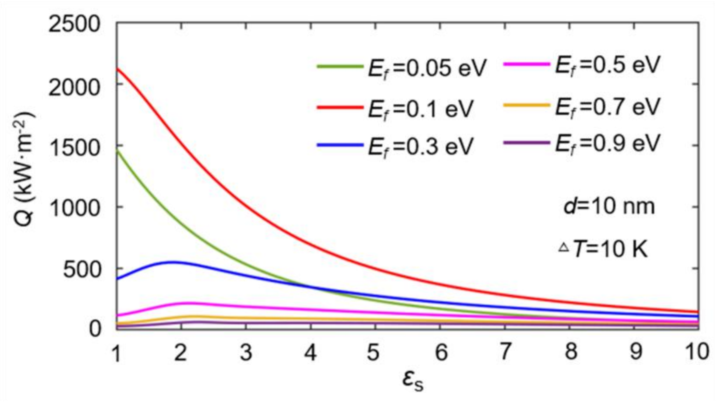

Figure S4. Radiative heat flux between two sheets of Bi_2Se_3 varies with the permittivity of the substrate at different Fermi energies. Related to Figure 6.

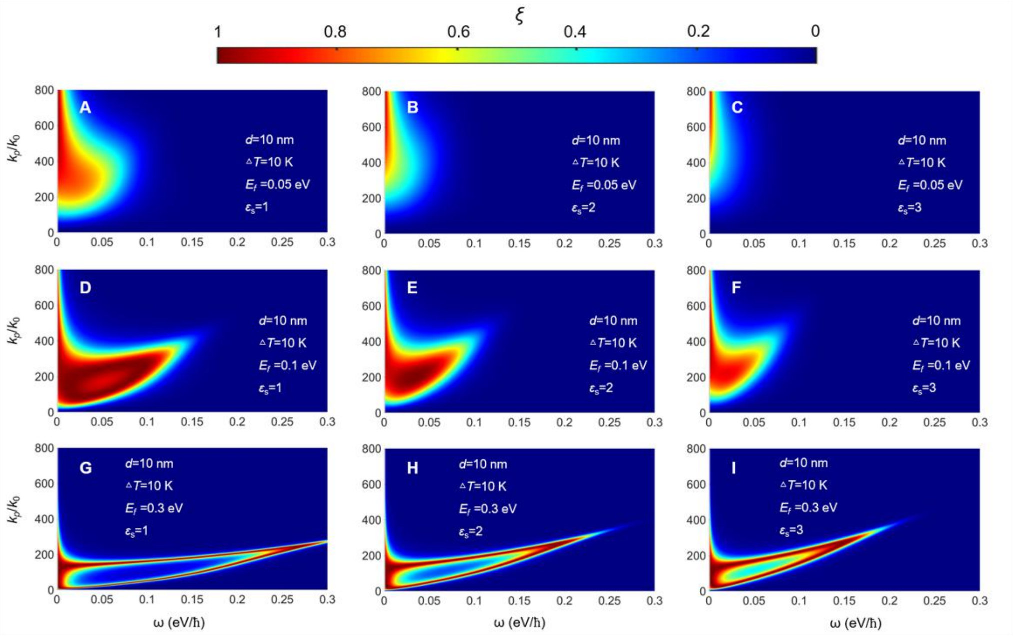

Figure S5. The photonic transmission coefficient for different cases: (A) E_f =0.05 eV, ε_s = 1, (B) E_f =0.05 eV, ε_s = 2, (C) E_f =0.05 eV, ε_s = 3, (D) E_f =0.1 eV, ε_s = 1, (E) E_f =0.1 eV, ε_s = 2, (F) E_f =0.1 eV, ε_s = 3, (G) E_f =0.3 eV, ε_s = 1, (H) E_f =0.3 eV, ε_s = 2, and (I) E_f =0.3 eV, ε_s = 3. The vacuum gap is d = 10 nm. Related to Figure 6.

Data S1. Matlab code for calculating near-field radiative heat transfer

Related to STAR Methods.

code1-“Main program”

```
clear all
% Physical parameters
h = 1.054571596e-34;
global c0;
c0 = 2.99792458e+8;
kb = 1.3806503e-23;
u0=4*pi*10^-7;
ep0=8.854*10^-12;
qe = 1.602176462e-19;
T_h0=[310];
T_l=[300];
ni=[100]*1e13*1e4;
T=300;
darray1=[10]*1e-9;
EE0=[0];
t=50*1e-9;
% Initiaization of different parameters
w1=0.001*(qe/h);
w2=0.3*(qe/h);
dw=0.0001*(qe/h);
nw = floor((w2-w1)/dw+1);
%calculation of total energy flux
NN=length(darray1)*length(ni)*nw;
N=0;
for i=1:length(darray1)

        Th=T_h0;
```

```
d1=darray1(i);

EE=EE0;

for j=1:length(ni)

        d2=d1;

        Tl=T_l;

        index=0;

        Ef=0.09;%sqrt(ni(j)*pi)*1e6*h/qe;

        eps=10;

        for w=w1:dw:w2

            N=N+1;

[sigma]=Bi2Se3(w,Ef*qe);

index = index+1;    fre(index)=w;

ae1 = (w+1)/c0;

ae3 = 2000*w/c0;

nke0 = 2000;

erre0 = 1.0;

Q1=h*w/(exp(h*w/(kb*Th))-1);        %% Planck Oscillator

Q2=h*w/(exp(h*w/(kb*Tl))-1);

Q=power(h*w,2)*exp(h*w/(kb*T))/(kb*power(T*(exp(h*w/(kb*T))-1),2));

  [vale0,se(index,:),kx0,] =simpson_e(w, nke0, d1, ae1, ae3,sigma,eps);

kx=kx0/(w/c0);

flux0(index)=vale0*(Q1-Q2);

F(i,index)=flux0(index);

        end

Q_t0(i,j)=(flux0(1)+4*sum(flux0(2:2:(nw-1)))+2*sum(flux0(3:2:(nw-2)))+flux0(nw))*dw/3;

end

end

plot(fre*(qe/h)^(-1),F*10^9)
```

code2-“Bi2Se3”

```
function [sigma,t]=Bi2Se3(w,Ef)
qe = 1.602176462e-19;
h = 1.054571596e-34;
miu=600;%cm2 V-1 s-1
vf=0.5*1e8;%cm/s
t=miu*Ef/(qe*vf*vf);%s
ni=2*(Ef/(vf*h))^2/(4*pi);%cm-2
sigma=1i*qe*qe*Ef/(h*h*4*pi*(w+1i/t));
end
```

code3-“simpson_e”

```
function [Int_val,se,kx] = simpson_e(w, n, d, min, max,sigma,ep)
        n1 = n;
        [s_e,dkx,se,kx] = func_e(w, n1, d, min, max, sigma,ep);
        temp2=(s_e(1)+4*sum(s_e(2:2:n1))+2*sum(s_e(3:2:n1-1))+s_e(n1+1))*dkx/3;
                Int_val = temp2;
function [s_e,dkx,se,kx] = func_e(w, n, d, min, max, sigma,ep)
global c0
dkx = (max-min)/n;
kx = zeros(n+1,1);
s_e = zeros(n+1,1);
u0=4*pi*10^-7;
ep0=8.854*10^-12;
    for ind=1:n+1
        kx(ind)= min+(ind-1)*dkx;
        kz= sqrt(ep*w*w/(c0*c0)-kx(ind)^2);
            kz0 = sqrt(w*w/(c0*c0)-kx(ind)^2);
```
